# Psychometric Properties of the Persian Version of Self-Management Scale for a Sample of Iranian Patients With Epilepsy

**DOI:** 10.5812/nms.10309

**Published:** 2013-06-27

**Authors:** Nahid Dehghan Nayeri, Mansooreh Aliasgharpour, Mohammad Ali Yadegari

**Affiliations:** 1 Nursing and Midwifery Care Research Centre, School of Nursing and Midwifery, Tehran University of Medical Sciences, Tehran, IR Iran; 2 Department of Medical-Surgical Nursing, School of Nursing and Midwifery, Tehran University of Medical Sciences, Tehran, IR Iran; 3 Mosavi Hospital, Zanjan University of Medical Sciences, Zanjan, IR Iran

**Keywords:** Psychometric, Factor Analysis, Epilepsy, Self Care

## Abstract

**Background:**

Despite the importance of self-management in epileptics, no instrument has been developed or validated in Iran. Since self-management is a multi-dimensional construct, having a valid and reliable instrument for measuring this compound construct is crucial.

**Objectives:**

This study aims to validate the Persian version of the self-management scale and provide a valid and reliable tool to measure self-management of patients with epilepsy.

**Patients and Methods:**

This is a methodological psychometric study. Construct , face and content validity was calculated on 200 samples after translation. Tool reliability was examined by using two methods: internal consistency and test-retest. Finally, the modified model was presented using exploratory factor analysis for the Iranian version of the tool.

**Results:**

The validity of all items was above 0.63 and their content validity indexes (0.81-1) were appropriate. Construct validity, exploratory and confirmatory factor analysis confirmed all the dimensions except for some safety and pharmacotherapy items. The overall tool reliability with internal consistency had alpha of 0.77.

**Conclusions:**

Persian version of the self-management scale for patients with epilepsy is valid and reliable to measure the dimensions of self-management in Iranian patients and it can be used to measure epileptics’ self-management. Further research on the safety of this tool is recommended.

## 1. Background

Epilepsy is a common chronic neurological disease after headache (1) and 2.4 million people are added to the number of epileptics in the world every year. This rate is higher in developing countries; so that about ¾ of people with epilepsy live in developing countries (2-5). Epilepsy interferes with the daily lives of patients. In addition to the social and economic burden, it leads to loss of productivity. Patients spend a lot of money on medical and traditional treatments (5). Moreover, compared to the general population; epileptics are 2.6 times more at the risk of premature death (6-8). Therefore, self-management of these patients is very essential; in a way that 70-80% of patients can have a normal life with proper treatment, which can be achieved with self-management (3, 9). Self-management is a multi-dimensional construct. It refers to actions that people should take for controlling chronic diseases such as epilepsy (10, 11). Assessing patients with chronic disease and their abilities in overcoming the critical condition is considered as nurses’ responsibilities. Also, having precise tools of measurement is of the prerequisites of appropriate intervention for this group of patients. Since 1992, DiIorio et al. have started a series of studies regarding self-management in patients with epilepsy and developed a tool for measuring it. After making the primary 26-item tool valid and reliable, 12 more items related to life style and safety. Factor analysis revealed five aspects of medication, information, seizures, safety and life style (6, 10). Although the validity of the self-management tool for medication in children with epilepsy has been conducted by Moody et al. in 2010 (12), all aspects of self-management in epileptic adults have not been studied in previous studies and researchers found that this tool has not been psychometrically evaluated in any country other than its country of origin. Given the importance of self-management in adult patients with epilepsy, it is necessary to validate the self-management scale to give epileptic patients more control over their lives. In order to use a questionnaire in other cultures, it should be translated, and retranslated. Moreover, the translated version should be adapted to the original version and culturally modified. At the end, if a question is ambiguous, it should be improved (13).

## 2. Objective

This study aims to psychometrically evaluate "self-management scale for patients with epilepsy" and was conducted on a sample of Iranian epileptic patients.

## 3. Patients and Methods 

The present study is a methodological psychometric research. The tool that was used in this study is the self-management scale for patients with epilepsy that was developed by DiIorio et al. (6, 10). The tool contains 38 items in a five choice Likert scale format with the scores ranging from one to five. Score of one indicates that the patient does not use self-management for that item. Score of 5 shows that patient always uses self-management for that item. Aspects of this instrument are medication management (10 items), Information (8 items), safety (8 items), seizures (6 items) and lifestyle (6 items). Possible scores range were respectively from 10 to 50 in medication, 8-40 in Information, 8-40 in safety, 6-30 in seizure and 6-30 in lifestyle subscales. Overall range of possible scores is 38 to 190 considering the total number of items. Higher scores indicate higher levels of self-management. The following steps were performed for tool psychometrics:

1. For the purposes of translation and back-translation of the questionnaire we used forward-backward method. Two native experts first translated the main tool into Persian. A neurologist and a researcher who were fluent in English separately did the reverse translation into English. After a series of discussions, they agreed upon translations of all items. In the next step, three researchers evaluated the Persian version and a number of items that did not match Iranian culture and language were modified (For example, there was an item about a lot of alcohol consumption and the word “a lot of” was removed from the Persian version). At last final version of the translated Persian was developed.
2. After the translation was completed, the instrument was given to 11 experts to work on its content validity. In order to achieve content validity, the experts were asked to comment on reasonability, suitability, attractiveness and logical sequence of items, as well as conciseness and comprehensiveness of the tool. Then, Content Validity Ratio (CVR) and Content Validity Index (CVI) of the tool were assessed. These two indicators are the most commonly used quantitative methods to determine the content validity of multiple choice questionnaires. The CVR indicates necessity and CVI indicate relevance, simplicity and clarity of questions according to the experts (14).
3. To determine the content validity of each item, we used three spectra: "It is necessary", "it is helpful but not necessary", "not necessary". Using the formula, the experts compared the achieved scores with Lawsche table. Accepted cut-off point of 11 experts was 0.05 (equivalent to 0.59) (15). In order to calculate the CVI for each item, features like "being simple and clear" were taken into account using four-choice Likert scale from not clear up to very clear and not simple to very simple (16, 17). After content validity confirmed, in order to achieve face validity, the tool was given to 10 patients with epilepsy to assess it in terms of simplicity, readability and clarity. Items that were difficult to understand were simplified and modified. To assess the construct validity of the tool, exploratory and confirmatory factor analysis techniques were used. For factor analysis, the available sample of eligible patients at Zanjan Valiasr Hospital and Epilepsy Association of Tehran were assessed in 2012. According to the literature for factor analysis, the minimum ratio of items to subjects should be about one to five (18). Therefore, 200 patients recruited. After obtaining informed consents from all 200 patients, they were asked to answer the self-management scale. Inclusion criteria for this study were: age between 15 and 70, having a medical diagnosis of epilepsy, taking antiepileptic drugs during the last year and having at least one seizure. If patient was illiterate, the tool was filled by interview. We used Statistical Package for Social Sciences (SPSS) software version 16 for exploratory factor analysis such as: Barltlet specificity test for evaluating adequacy of factor analysis, Principal Axis Factoring method for extracting the factors (constructs), method of Varimax rotation (Kaiser normalization with zero delta), Scree plot and Eigenvalue greater than one for component rotation. Also a load factor of at least 0.3 was considered for the items. After exploratory factor analysis, in order to assess the factors obtained from this analysis there was a need to fit the model with confirmatory factor analysis to identify the following features: Whether the proposed theoretical model is confirmed? And are the defined coefficients of the model significant? Lisrel 8.8 software was used for this purpose. The maximum likelihood method was used for model fitting and variance covariance matrix of data was used as input into the model. After confirming the theoretical model based on data, we used the model to assess defined relationships in terms of significance and evaluating research hypotheses. T-value index was used in order to assess the significance of the model coefficients. If the index value is greater than 1.96, the coefficient is statistically significant at the level of 0.05 (19, 20). Reliability: Cronbach’s alpha was used in order to investigate the internal reliability of the questionnaire. To investigate the stability, the test-retest method was used by calculating Intra-Class correlation coefficient for 30 individuals with two-week intervals. The reliability of the tool was measured in all aspects.

### 3.1. Ethical Consideration

This paper is a report of research project approved and funded by Tehran University of Medical Sciences (TUMS). Ethics committee of TUMS confirmed all process of the study. After approval of TUMS, and the respected medical centers, we started the study. We explained goal and objectives of the study to all of participants. Then those who were willing to participate in the study signed an informed consent. Also they were free for withdraw from the study.

## 4. Results

### 4.1. Description of Samples

The study involved 200 patients with epilepsy; half of the participants (50.5%) were aged between 18 to 25 years old. In total, 43.5% of participants were females and 56.5% were male. Also, 57% were single, more than one third (41.5%) were unemployed. In total, 44.5% were under 16 years old when their seizure started and, 52% were taking anti-seizure medication, and Seizure type of the majority of participants (92%) was the general type (Table 1).

**Table 1.** Demographic Characteristics of Patients with Epilepsy

Demographic Characteristics	Number (%)
**Age, y**	
18-25	101 (50.5)
26-35	56 (28)
Older than 36	43 (21.5)
**Gender**	
Female	87(43.5)
Male	113 (56.5)
**Marital status**	
Married	82 (41)
Single	118 (59)
**Employment status**	
Employed	68 (34)
Retired	7 (3.5)
Unemployed	83 (41.5)
Housewife	42 (21)
**Age seizure started, y**	
Under 16	89 (44.5)
17-30	84 (42)
31-45	21 (10.5)
Over 46	6 (3)
**Number of medications**	
Single	104 (52)
Multi	96 (48)
**Type of seizure**	
Focal	16 (8)
Generalized	184 (92)

### 4.2. Findings of Content Validity Index

CVR of the items were all above the cut-off values presented in Lawsche Table (0.59) and therefore all items were retained for the next stage. Also based on the results of CVI calculation, all the items were accepted, since their CVI values were greater than 0.81 (Table 2).

**Table 2.** CVR and CVI of Items of Questions Epilepsy Self-Management Scale

Number of Item	CVR^a^	CVI^a^
1	0.81	0.9
2	1	1
3	0.81	0.9
4	0.81	0.81
5	0.81	0.81
6	0.81	0.9
7	0.81	1
8	0.81	0.9
9	0.81	1
10	1	1
11	0.81	0.9
12	0.81	0.9
13	0.63	1
14	1	1
15	0.81	0.9
16	0.63	0.9
17	1	1
18	1	1
19	0.81	0.81
20	0.81	0.9
21	1	1
22	1	1
23	0.81	0.9
24	1	1
25	0.81	0.9
26	1	1
27	1	1
28	1	1
29	0.81	0.9
30	1	1
31	0.81	1
32	0.81	1
33	1	1
34	1	1
35	1	1
36	0.81	0.9
37	1	1
38	1	1

^a^ CVI, Content Validity Index; CVR, Content Validity Ratio

### 4.2.1. Results of the First Stage of Factor Analysis: Exploratory phase

**Figure 1.**
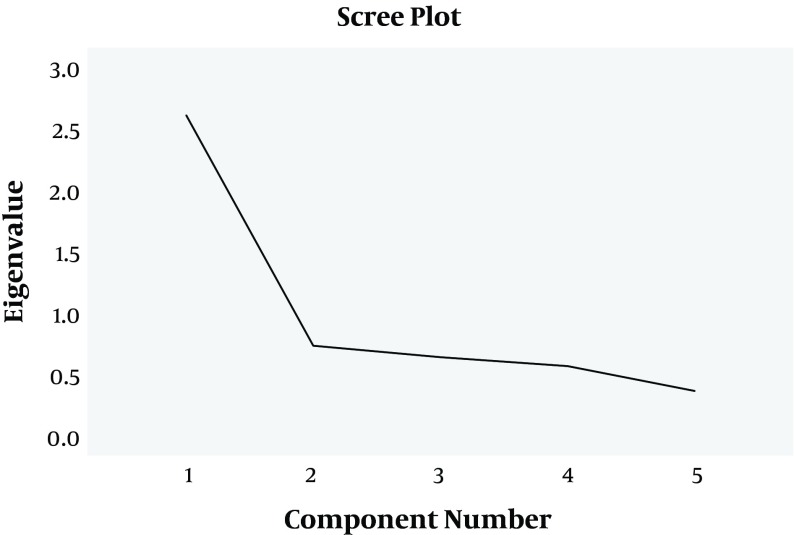
Scree Plot in the Exploratory Factor Analysis

In order to categorize the questions of instrument, a two-phase strategy was used; in the first phase, questionnaire items formed its structures. In the next phase, each of the structures formed epilepsy self-management indices. In the first phase, the index of Kaiser-Meyer-Olkin (KMO) test varied from 0.52 to 0.73, and the percentage of variance (TVE) changed between 18.73 and 35.37. In addition, Bartlett's test result (P < 0.001) confirmed adequacy of exploratory factor analysis for all the constructs. Load factor higher than 3.0 (criterion for selecting items) was observed in all the structures, except for item 5 from medication management and items 1 and 2 from safety. These items were up for elimination. During the second phase, KMO test result was 0.793 and percentage of explained variance was 52.668. Furthermore, Bartlett's test result confirmed adequacy of exploratory factor analysis. Scree plot test results that determine the number of factors at this phase are shown in Figure 1. The steep curve indicates and confirms the existence of final five factors for structures of the questionnaire. In other words, the 5-fold constructs eventually defined an ultimate construct. In this phase, results of specialized load factors were respectively 0.843 (self-management of seizures) and 0.675 (medication self-management). This confirms constructs coefficients in determining the ultimate factor.

### 4.2.2. Second Phase Of Factor Analysis: Confirmatory phase

In accordance with the strategy used in the exploratory factor analysis, second-order confirmatory factor analysis model was used. Chi index (2.03) confirmed the model fitting and RMSEA1 and RMSR2 verified validity of the model. However, IFI3 and CFI4 fitting indices were smaller than 0.9 and did not fully confirm the validity of this model. Consequently, based on this model we cannot confirm the structure resulting from exploratory factor analysis (Table 3).

**Table 3.** Fitting Indices, Assessing Suitability of Confirmatory Factor Analysis of the Modified Model

X^2^	X^2^/df	RMSR^a^	RMSEA^a^(95% CI^a^)	CFI^a^	IFI^a^
799.09	1.78	0.079	0.063 (0.055, 0.070)	0.90	0.90

^a^ CFI, comparative fit index; CI: Confidence Interval; IFI: Incremental Fit Index; RMSEA, root mean square error of approximation; RMSR, root mean square residual

### 4.2.3. Phase Three: Modify the Model by Exploratory Factor Analysis 

Since some of the items aspects were not quite significant in the confirmatory model, the model was modified to result in a significant final model. Safety self-management items (items 1, 2, 5, 7 and 8) and the medication self-management item 5 that were not noteworthy were removed from the model. After excluding these items, the model achieved a proper fitting level. The final Iranian model has five aspects. Table 3 shows the modified model parameters. Based on the presented index values for this model, and Chi2 index with its degree of freedom smaller than five (1.78) confirmed this model. RMSEA and SRMR indices, as well as IFI and CFI indexes which were greater than or equal to 9.0 also confirmed the validity of this model. Therefore, this model was relatively fitted to the desirable level. Based on this model, we confirmed the results of exploratory factor analysis.

### 4.2.4. Reliability of Instrument

In order to determine the reliability of the research tool after the modifications, we used Cronbach's alpha internal consistency test and concurrent reliability test-retest method. Calculated values and all aspects of the tool are presented in Table 4.

**Table 4.** Evaluation of Reliability of Items and the General Reliability

Factor	Interclass Correlation Coefficient In the final form	Cronbach’s alpha	No. of Items in The Iranian Questionnaire	Cronbach;s alpha of original Toll	No. of Items in The Original Questionnaire
**Information section**	0.788	0.652	8	0.688	8
**Life style section**	0.868	0.551	6	0.551	6
**Medication section**	0.810	0.73	9	0.716	10
**Safety section**	0.842	0.313	3	0.320	8
**Seizure section**	0.772	0.62	6	0.620	6
**Self-Management section**	0.850	0.847	5	0.770	5

## 5. Discussion

This study aimed to provide a valid and reliable instrument to measure self-management of Iranian patients with epilepsy. Although the validity of the self-management tool for medication in children with epilepsy have been conducted (12); this tool did not validate in epileptic adult patients unless in its country of origin. However, the original study was mainly conducted on young male people who had general type of epilepsy and seizure was mostly started in their adolescence. Other studies have shown higher prevalence of epilepsy in men too (21, 22). Also some studies reported more seizures in adults occur before the age of 20 (23). Results showed that the modified model for Iranian patients is adequately valid and reliable. The primary tool consisted of 38 items. However, after different stages of psychometric evaluations of construct validity phase, number of items was reduced to 32. Levels of content validity and CVI showed that all items of the tool are contently valid. Therefore, we kept all the items. This result was expected, since original tool developers not only reviewed all relevant literature, but also interviewed the patients and their professional caregivers. The basic form of exploratory factor analysis showed proper load factor (greater than 3.0) in all the items except for medication and safety self-management items. These items were later eliminated. In this step, the results of the analysis revealed 5 factors. This number is equivalent to the original questionnaire, which was introduced by Dilirio et al. This was confirmed by confirmatory factor analysis, so that the IFI and CFI indices were smaller than 0.9 and did not completely approve the validity of the model. It can be concluded that since, safety self-management items did not have desirable construct validity it weakened the validity of entire questionnaire. In correction phase, items without a proper load factor were eliminated. Inadequate load factors of these items seem to be due to cultural differences. For example, the occurrence probability of item "swimming alone" in Iran is low, as once a patient is diagnosed with epilepsy he/she is often accompanied by a family member all the time. The item "use of alcohol" is fundamentally different from the country of origin. Due to religious and cultural instructions, alcohol is prohibited especially after the diagnosis. In self-management of medication, an item (forgetting to take your medicine) was eliminated from the model due to insignificance. Regarding items of medication self-management, this may partly reflect differences in type and amount of medication to control seizures in patients from different countries (21, 24, 25). After excluding these items, the model achieved appropriate fitting level; so based on this model, the structure was confirmed by exploratory factor analysis. The Iranian validated instrument has the same number of items as the original version in sections of information self-management, self-management of seizures and lifestyle. The tool has 32 items in total; nine items in medication self-management and 3 items in safety. Excluded questions were questions number 1, 3, 7 and 8 in safety section. They were about “swimming alone," "going out and staying up late", "using electrical devices", "climbing chairs and ladders" and "drinking alcohol". Medication self-management question number 5 about "postponing to provide the medicine due to cost" was also deleted. The results of the two methods concerning the reliability of the tool indicated good reliability of the questionnaire as a whole. However, the questionnaire lacked reliability in some sections, which was acceptable due to low number of questions in each section. The main purpose of this study was using a valid psychometric tool for assessing Iranian patients’ self-management and improving their treatment by staff and nurses. Therefore, we can achieve good control of the disease. Findings of the study which was a psychometric validation of instrument for self-management of epileptic patients, have an important role in the first and most important step of the care process i.e. assessing the patient. This tool can be used in Iranian Epilepsy Association, clinics and medical centers in order to measure and effectively promote self-management of patients and to avoid wasting time and reduce patients’ health care costs. Given the lack of adequate reliability and validity of the safety section in this tool, further research in this area is recommended.
